# Willingness to Take COVID-19 Vaccines in Ethiopia: An Instrumental Variable Probit Approach

**DOI:** 10.3390/ijerph18178892

**Published:** 2021-08-24

**Authors:** Abayomi Samuel Oyekale

**Affiliations:** Department of Agricultural Economics and Extension, North-West University Mafikeng Campus, Mmabatho 2735, South Africa; abayomi.oyekale@nwu.ac.za

**Keywords:** COVID-19, viral strain, vaccine, vaccination, willingness, Ethiopia

## Abstract

This paper analyzed the factors influencing the willingness of Ethiopia’s population to take COVID-19 vaccines. The data included the COVID-19 High Frequency Phone Survey of Households in Ethiopia that were collected in 2021. This paper relied on the 10th round of the survey, which was comprised of 2178 households. The Instrumental Variable Probit regression model was used to analyze the data. The results showed that majority of the respondents (92.33%) would receiveCOVID-19 vaccines, while 6.61% and 1.06% were, respectively, unwilling and unsure. Across the regions of Ethiopia, Southern Nations, Nationalities, and Peoples’ Region (SNNPR) (99.30%), Oromia (97.54%), Tigray (97.04%) and Gambela (95.42%) had the highest proportions of respondents willing to have the vaccine. Vaccine safety concern was the topmost reason for those unwilling to receive the vaccine. The results of the Instrumental Variable Probit regression showed that currently working, age, engagement with non-farm businesses and region of residence significantly influenced the population’s willingness to take the vaccine (*p* < 0.05). It was concluded that although the willingness be vaccinated was impressive, without everyone being receiving the COVID-19 vaccine, infection risk can still be high; this is due to the persistent mutation of the viral strains. Thus, there is a need to intensify efforts toward addressing the safety issues of COVID-19 vaccines, while efforts to enhance acceptability should focus on the youth population and those who are unemployed.

## 1. Introduction

Judging by the intensity of its morbidity and mortality, COVID-19 has proved to be one of the worst pandemics the world has recently witnessed. Having been declared as a Public Health Emergency of International Concern (PHEIC), addressing COVID-19 requires the proper administration of medical services, among which effective vaccination cannot be over-emphasized [1]. This is in alignment with conventional wisdom derived from the fact that, aside from access to safe drinking water, no other interventions in the history of mankind have had significant impacts on reducing incidences of morbidity and mortality as vaccinations [2]. The World Health Organization (WHO) is therefore advocating for speedy interventions that would ensure access of every individual to COVID-19 vaccines, as a way of securing our collective existence given the severity of the ongoing pandemic.

Generally, vaccines are meant to enhance the immune system through their extraordinary ability in responding to and remembering certain encounters with pathogenic antigens [3]. More importantly, over the past few decades, a number of success stories in addressing many pandemics of worrisome morbidity and fatality have been directly associated with development and effective utilization of vaccines. It has been estimated that, annually, vaccines prevent about 6 million deaths that could have resulted from vaccine-preventable diseases [4]. Therefore, healthcare professionals and policymakers are now advocating for development of vaccines in sufficient quantities, in order to address the ongoing COVID-19 pandemic.

Vaccines can be classified as live or non-live. Live vaccines are used with certain restrictions since they can replicate uncontrollably in individuals whose immune systems are compromised as a result of underlying medical conditions [3]. However, individuals with compromised immune systems can access non-live vaccines without risk, although their efficacy may be substantially sub-optimal [5]. However, due to several factors, addressing COVID-19 through vaccination presents some peculiar challenges. The first borders on several speculations on the emergence of the virus, many of which may influence acceptability of global efforts in addressing its spread. Specifically, there have been a number of conspiracy theories advancing the hidden international agendas throughout the COVID-19 saga, many of which are precipitating fears and vaccine hesitancy [6].

The emergence of social media as major platform for information-sharing has rendered an individual of the 21st century more informed, whether rightly or wrongly. Specifically, it should be reemphasized that the acceptability of polio vaccines in Nigeria, Pakistan and Afghanistan was affected by misinformation on its tendency to inhibit fertility [7,8]. Presently, the information around COVID-19 vaccines has been diverse, and much of this information is spreading misleading rumors [9]. There have been underlying notions that COVID-19 vaccines may contain deliberate poisons that are meant to control global population [10]. In a recent study, 91% of the information obtained from several countries on the internet was classified as rumors [10]. These include the fear of being sick or dying after receiving the COVID-19 vaccination and that the vaccine is a messenger Ribonucleic acid (mRNA) that may distort the sequence of human deoxyribonucleic acid (DNA), thereby turning them into mere prototypic beings whose genetic compositions may have been silently modified [10].

Several factors may influence an individual’s conception or belief in medical procedures for disease prevention. People also the fundamental right to decline whatever options are presented to them by healthcare professionals [11]. Except by legislative means, many countries cannot force individuals to be vaccinated against their will. More importantly, some authors have highlighted the factors that can influence the decision to comply with government’s vaccination programs. These include possession of proper education on health-related issues, an individual’s perception of the level of risk [10,12,13,14], race [15], perceived risk of infection [16], perceived benefits [17], age [15,18,19,20,21], educational level [18], gender [15,19,21], chronic medical condition [19], access to health insurance [19], ethnicity, income and employment [22] and previously being tested for the virus [18].

It should be emphasized that most of these studies have been conducted in the United States of America and China. There are currently very few studies on African countries and Ethiopia in particular. Studies on the willingness to partake in the COVID-19 vaccination program are not only important but essentially timely. Such studies can enhance the understanding of health policymakers on socio-economic and demographic factors that influence vaccination compliance. This is going to assist public health practitioners in evaluating the expected effectiveness of resources that are being committed to the procurement of vaccines and the expected impacts on the control and management of COVID-19 pandemic.

## 2. Materials and Methods

### 2.1. The Data

This study used the data from the COVID-19 High Frequency Phone Survey of Households in Ethiopia that commenced in 2020 [23]. The World Bank leveraged the Living Standards Measurement Study (LSMS) to implement this survey based on existing long-term collaboration with the Central Statistical Agency (CSA) of Ethiopia [23].The survey is therefore a subsample of the Ethiopia Socioeconomic Survey (ESS) that was conducted by the CSA in 2018/2019. ESS is a long-term project that is being implemented by the CSA, which is constitutionally mandated according to Ethiopia’s Proclamation No. 442/2005 to coordinate the National Statistical System (NSS) in order to provide time-responsive datasets for informed policy decisions [24].

The survey was telephonically conductedbetween 1st and 23rd of February 2021 and the World Bank has implemented similar collaborations between national statistical agencies of five other African countries, including Tanzania, Malawi, Nigeria, Uganda and Kenya [23]. The respondents were contacted through the phone numbers that were provided during the previous ESS. The sampling frame was therefore formed by the list of all the households that supplied their phone numbers, because correctly addressing the COVID-19 pandemic necessitates social distancing and intermittent lockdowns within Ethiopia [25].

Specifically, out of the 7527 households that formed the sampling frame for wave 4 of the 2018/2019 ESS [26], valid phone numbers were obtained for 5374 households, with 4626 being phone-owners and 995 providing reference phone numbers. The first round of the survey targeted 3300 households for an interview in order to ensure national representativeness. Specifically, 1300 rural households and 2000 urban households were targeted. It should also be noted that 1413 rural households had phones and 771 provided reference phone numbers. Additionally, 3213 urban households had phones and 224 could be reached through reference phone numbers.

In the first round, the survey was implemented by calling all of the phone numbers that were provided in order to account for non-response and attrition [25]. A household is flagged as non-response only after being called at least three times in three days [25]. The consent to participate in the survey was sought from those who were reached on their phones after the objectives of the survey had been clearly explained to them. Most of the respondents were the heads of the household. However, in few cases where the heads were indisposed, a representative with adequate information on the contents of the questionnaire stood for them.

The survey used the modular approach of the Computer Assisted Telephone Interview (CATI) techniques. The questionnaire was loaded with Survey CTO (Dobility, Inc., Cambridge, MA, USA). This is a CATI software that has been noted as one of the most reliable software for collecting data in offline settings [23]. Tablets were given to every enumerator along with sufficient data bundles which were loaded on their personal mobile phone devices. The collected data were daily sent to the central server and Senior Field Supervisors were designated to review the survey with the enumerators twice-a-day through phone calls in order to address quality concerns. Audio recordings of the interviews were also logged in order to reconcile outliers or non-response issues [23]. The interviews were carried out in six languages: Wolayita, Afan Oromo, Amharic, Afar, Somali and Tigrigna [25]. During the first round in 2020, 3249 households were successfully interviewed (2271 from urban areas and 978 from rural areas). At each round of data collection, the consents of the interviewed households were sought for inclusion in the next survey [25]. However, during the 10th round, only 2178 households completed the survey, with 537 from rural areas and 1641 from urban areas.

### 2.2. Limitations of the Data

The survey suffers from limitations because intermittent lockdowns prevented the conduction of face-to-face interviews with the respondents. In addition, using a sampling frame that was based on those whose phone numbers or references phone numbers were provided during the 2018/2019 ESS also undermines the representativeness of the data. This is a result of the low penetration of telecommunication in rural Ethiopia. This is more evidently revealed by the low proportion of rural respondents in the dataset, unlike the results from ESS that were conducted via face-to-face interviews in 2018/2019 [26]. Dropout of respondents, as characterized by panel data, is also a matter of concern. Specifically, the first round of the survey had 3249 respondents, compared to 2178 in the tenth round.

### 2.3. Instrumental Variable Probit Model

The Instrumental Variable Probit regression model was used to analyze the data. Conventionally, the Probit model is used when the dependent variable is binary in nature. The Probit model uses the cumulative Gaussian normal distribution function to calculate the probability of belonging to any of the categories.The standard Probit model is not applicable for this study because of the suspicion that the “currently working” variable could be endogenous, thereby implying that the estimated parameters would be biased. Therefore, an extension of the Probit model, in the form of the Instrumental Variable Probit regression, was used. The model is ideal for a situation whereby a dichotomous dependent variable, estimated as dummy variable, is suggested to be influenced by some independent variables, among which there is a variable that had been suspected to be endogenous [27].

(1) $$Y_{i}=\alpha +\beta_{k}\overset{v}{\underset{k=1}{\sum }}X_{ik}+\delta W_{i}+\varepsilon_{i}$$

(2) $$W_{i}=\mu +\varphi_{k}\overset{z}{\underset{k=1}{\sum }}X_{ik}+\pi G_{i}+\epsilon_{i}$$

In Equation (1), the dependent variable ($Y_{i}$) was coded as 1 for willing to take COVID-19 vaccines and 0 (0) for otherwise. $X_{ik}$ are the explanatory variables which include urban area (yes = 1, 0 otherwise), occupation—unemployed is the base variable—[agriculture (yes = 1, 0 otherwise), industry/manufacturing (yes = 1, 0 otherwise), wholesale and retail trade (yes = 1, 0 otherwise), transport services (yes = 1, 0 otherwise), restaurant (yes = 1, 0 otherwise), public administration (yes = 1, 0 otherwise), personal service like beauty salon (yes = 1, 0 otherwise), construction (yes = 1, 0 otherwise) and education/health (yes = 1, 0 otherwise)], regional variables—Tigray is the base variable—[Afar region (yes = 1, 0 otherwise), Amhara (yes = 1, 0 otherwise), Oromia (yes = 1, 0 otherwise), Somali (yes = 1, 0 otherwise), Benishangul-Gumuz (yes = 1, 0 otherwise), SNNPR (yes = 1, 0 otherwise), Gambela (yes = 1, 0 otherwise), Harar (yes = 1, 0 otherwise), Addis Ababa (yes = 1, 0 otherwise) and Dire Dawa (yes = 1, 0 otherwise)], age of household head (years), farming income (yes = 1, 0 otherwise) and business income (yes = 1, 0 otherwise). $W_{i}$ is the endogenous explanatory variable coded as 1 for currently working and 0 (0) otherwise. Equation (2) estimates the determinants of being currently working with gender of the respondents$\;(G_{i})$, which was coded as 1 for males and 0 otherwise representing the instrumental variable. If $W_{i\;}$ is not endogenous, then $Cov\left(X_{i}\varepsilon_{i}\right)=0$. However, if this is violated, estimating Equation (1) with standard a Probit regression model will produce biased and inconsistent parameters [28]. The analyses for this study were carried out with STATA 13 software. The software generated the Wald test of exogeneity statistic, which, if shown to be statistically significant, implies the adequacy of the instrumental variable(s) and the use of IV Probit is thus justified.

## 3. Results

### 3.1. Socioeconomic Characteristics of Respondents

Table 1 shows the distribution of the respondents’ socioeconomic factors based on their decision to receive the vaccination. It shows that majority of the respondents (92.33%) would get a COVID-19 vaccination if the approved vaccines are freely available. The results in the Table further reveal that respondents from urban areas accounted for 75.34% of the respondents and majority of those who were not willing to be vaccinated came from urban areas. Based on gender, 62.21% of the respondents were males, but more females were unwilling to be vaccinated. In addition, 69.65% of the respondents were working at the time of the survey. The majority of the respondents were in their active age brackets of 25 < 45 years, while 4.64% were over 59 years of age.

**Figure 1:**
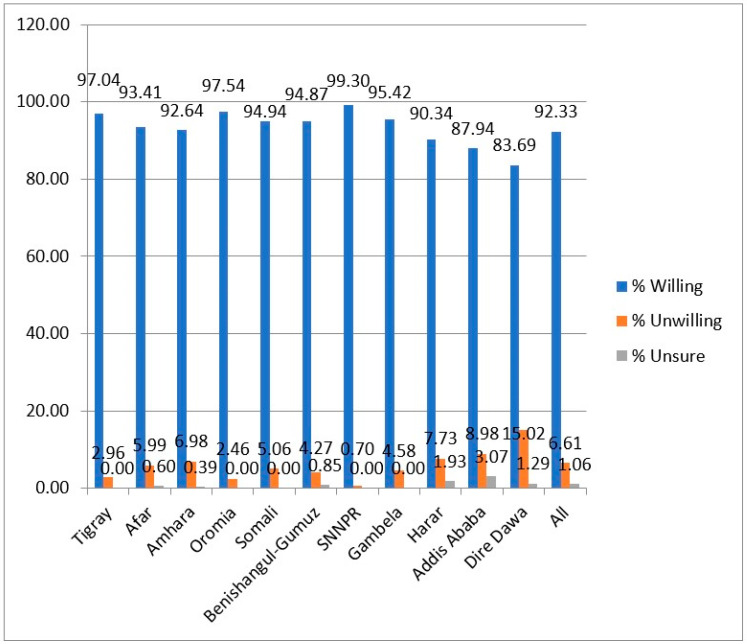
Distribution of respondents based on willingness to get COVID-19 vaccines across the Ethiopia’s regions.

**Figure 2:**
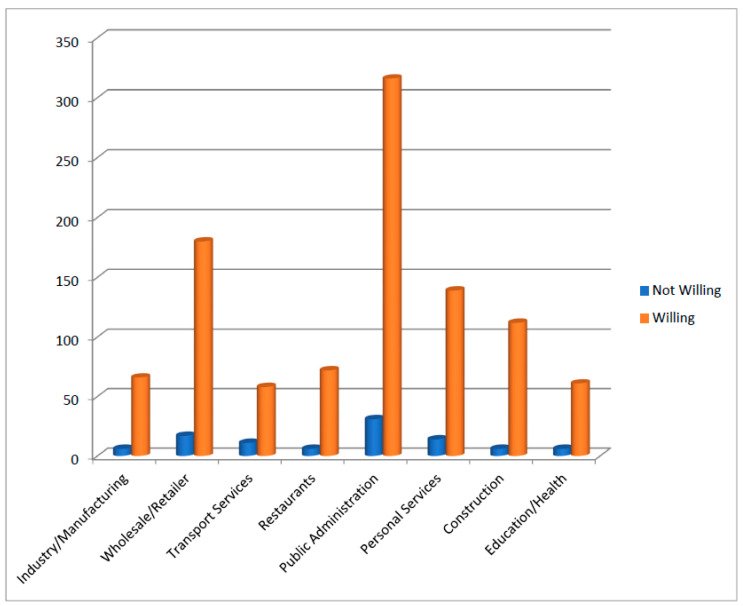
Frequencies of respondents willing to get COVID-19 vaccines across occupational groups in Ethiopia.

**Figure 3:**
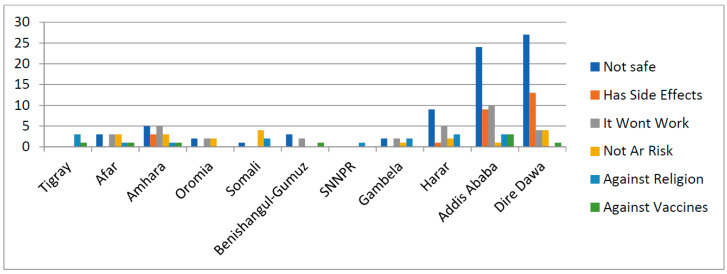
Frequencies of respondents’reasons for not willing to get COVID-19 vaccines across the regions in Ethiopia.

Figure 1 and Figure 2 further reveal the distribution of the respondents’ decisions to get a COVID-19 vaccine across different regions and occupational groups in Ethiopia. Figure 1 shows that SNNPR (99.30%), Oromia (97.54%), Tigray (97.04%) and Gambela (95.42%) had the highest proportions of vaccine-willing respondents while Dire Dawa (83.69%), Addis-Ababa (87.94%) and Harar (90.34%) had the lowest percentages. Figure 2 also shows that the respondents who occupied public administration positions constituted the highest frequencies of those that were willing and unwilling to be vaccinated. Figure 3 presents the reasons that were provided by the respondents for being unwilling to be vaccinated, coming from the different regions across Ethiopia. This Figure reveals that the safety of the vaccines was the topmost reason provided by the respondents across the different regions. In the Dire Dawa region, however, the second most important reason that was provided for the rejection of COVID-19 vaccine was based on side effects it might have, while second most important factor in Addis Ababa was the fear that it would not work.

### 3.2. IV Probit Model Results

Table 2 presents the results of data analysis with the Instrumental Variable (IV) Probit regression approach. The model produced a good fit for the data based on the statistical significance (*p* < 0.01) of the computed Chi-square statistics (101.75). Additionally, the use of the Instrumental Variable Probit was justified by the statistical significance of the computed Wald test of exogeneity (*p* < 0.05). This implies that the currently working variable was truly endogenous and the instrumental variable was adequate.

The results further show that the parameter of the “currently working” variable is statistically significant (*p* < 0.01). This result implies that respondents who were currently working were far more likely to be willing to receive the vaccine. The parameter of age is also showed a positive sign and was statistically significant (*p* < 0.01). This also implies that old respondents were more likely to have a COVID-19 vaccine. The parameter of non-farm businesses showed a negative sign and was therefore statistically significant (*p* < 0.05). This shows that those respondents whose households opened non-farm businesses some four weeks before the survey had a significantly lower probability of receiving a COVID-19 vaccine. Additionally, the regional variables for Afar, Amhara, Somali, Harar, Addis Ababa and Dire Dawa showed a negative sign and were also statistically significant (*p* < 0.05). These results imply that compared with respondents from Tigray, residents from Afar, Amhara, Somali, Harar, Addis Ababa and Dire Dawa were less likely to have a COVID-19 vaccine.

## 4. Discussion

The results of data analyses show that most of the respondents (92.3%) indicated that they would be willing to have a COVID-19 vaccination if approved vaccines are available. This represents a large proportion of the population given that in some previous studies, lower proportions had been reported. Specifically, in the USA, 75% reported a willingness to be vaccinated [15]; in China, 28.7% indicated yes and 54.6% said probably [17]; in a survey in Philadelphia, 63.7% of employees indicated yes and 26.3% were unsure [18]. In Africa, 71% [29] and 59% [30] were willing to be vaccinated in South Africa and the Democratic Republic of the Congo, respectively. However, given the high-mutant property of COVID-19, a higher vaccination coverage would likely guarantee the attainment of herd immunity. This is due to a recent emergence of different variant strains of COVID-19 due to a progressive mutation that is rendering certain developed vaccines to be less effective [31,32].

The results further revealed that a higher proportion of urban dwellers were unwilling to be vaccinated compared to rural dwellers. However, the parameter of urban residence did not significantly influence the probability of willingness to receive a COVID-19 vaccine. This is expected because urban residents may have access to a high level of misinformation on the COVID-19 vaccine [9,12,13]. The results also show that more males were willing to be vaccinated than their female counterparts. This result reveals the gender factor that can impact efforts by government in addressing COVID-19 in Ethiopia. In some previous studies [15,19,21], gender was found to influence decisions around the COVID-19 vaccination. 

Older respondents were also far more likely to be willing to have a COVID-19 vaccine. This is expected because age is one of the major factors affecting COVID-19 infection, with elderly people having weaker immune systems and thus at a higher risk of infection. A similar finding had been reported in some previous studies [15,18,19,20,21]. Furthermore, the “currently working” variable significantly increased the probability of willingness to be vaccinated. This can be explained by the desirability of good health for individuals that are working, since being sick with COVID-19 would reduce their productivity and thus their income. However, the results show that engagement with non-farm businesses reduced the probability of willingness to have a COVID-19 vaccine. This points to the fact that self-employed individuals may be less likely to see the need to get COVID-19 vaccines because they are not subjected to the operational contents of any formal contracts. The results also indicate that willingness to get vaccinated is influenced by some regional variables. This reemphasizes the need to factor in regional differences when addressing the COVID-19 pandemic in Ethiopia.

The reasons for individuals’ unwillingness to get COVID-19 vaccines were also revealed. Specifically, pertinent issues surrounding the safety and the side effects of the vaccines were of the topmost concern to the respondents. In certain previous vaccination interventions that were meant to address prior epidemic outbreaks, safety had been a fundamental determinant of acceptance. Good examples include the polio vaccination in northern Nigeria [10], the H1N1 vaccine in Sweden and Finland [32], and the measles, mumps and rubella (MMR) vaccine in the United Kingdom and United States [33].

## 5. Conclusions

Understanding the expected behavior of the population in relation to the uptake of COVID-19 vaccination is a vital subject demanding thorough investigations and analyses. This is now more important, given the intermittent upsurge of infections, in what have been described as viral waves in many African countries. The consensus among policymakers is that addressing COVID-19 requires adequate vaccination of the entire population, based on the speed of infections, seriousness of morbidity and mutative features of the virus that are now undermining the effectiveness of the developed vaccines.

Based on the findings of this study, some recommendations can be made. It should be emphasized that several socioeconomic variables are of fundamental relevance in addressing the willingness to have a COVID-19 vaccine. Specifically, although older people would be more willing to receive COVID-19 vaccines, efforts to address the pandemic also require younger individuals to take adequate protective responsibilities, especially because they tend to be asymptomatic. In addition, there are some gender issues that should be taken cautiously in the ongoing efforts at promoting the effective management of COVID-19. Specifically, Ethiopian women are to be targeted with information to facilitate their responsiveness to COVID-19 vaccination. It is also important to ensure that occupational factors that are relevant for promoting COVID-19 acceptance in Ethiopia are addressed, understanding that informally engaged people would need to be specifically targeted with compelling information to address their hesitancy around the vaccine.

Furthermore, ongoing research efforts and international initiatives to provide workable vaccines against COVID-19 are commendable. However, more efforts are still needed in addressing the effectiveness of vaccines with significantly minimized side effects. Meanwhile, the provision of honest communication by healthcare practitioners on effectiveness and expected side effects of available COVID-19 vaccines would go a long way in helping individuals to make informed decisions on being vaccinated.

## Figures and Tables

**Table 1:** Respondents’ Demographic Characteristics.

Variables	Not Willing/Not Sure		Willing		Total	
Sector	Freq	%	Freq	%	Freq	%
Urban	148	6.80	1493	68.55	1641	75.34
Rural	19	0.87	518	23.78	537	24.66
Gender						
Female	93	4.27	730	33.52	823	37.79
Male	74	3.40	1281	58.82	1355	62.21
Currently Working						
No	51	2.34	610	28.01	661	30.35
Yes	116	5.33	1401	64.33	1517	69.65
Age of Respondents						
<20	3	0.14	38	1.74	41	1.88
20 < 25	21	0.96	183	8.40	204	9.37
25 < 30	39	1.79	366	16.80	405	18.60
30 < 35	24	1.10	342	15.70	366	16.80
35 < 40	33	1.52	321	14.74	354	16.25
40 < 45	24	1.10	371	17.03	395	18.14
45 < 50	6	0.28	132	6.06	138	6.34
50 < 55	9	0.41	96	4.41	105	4.82
55 < 60	5	0.23	64	2.94	69	3.17
>=60	3	0.14	98	4.50	101	4.64
Total	167	7.67	2011	92.33	2178	100.00

**Table 2:** Determinants of Willingness to Take COVID-19 Vaccines.

	Coef.	Std. Error	z	*p* > |z|
Currently working	0.9390874	0.257606	3.65	0.000
Regional variables				
Afar	−0.6098229	0.2523096	−2.42	0.016
Amhara	−0.8008101	0.246855	−3.24	0.001
Oromia	−0.2267666	0.2648143	−0.86	0.392
Somali	−0.6279075	0.3102659	−2.02	0.043
Benishangul-Gumuz	−0.4412474	0.2782665	−1.59	0.113
SNNPR	0.1603359	0.3947655	0.41	0.685
Gambela	−0.5025854	0.2848655	−1.76	0.078
Harar	−0.7888761	0.238634	−3.31	0.001
Addis Ababa	−0.8102018	0.2170748	−3.73	0.000
Dire Dawa	−1.010384	0.2244206	−4.50	0.000
Urban	0.1693273	0.1227028	1.38	0.168
Age of Respondents	0.0104907	0.0031925	3.29	0.001
Non-farm business	−0.2161269	0.1026179	−2.11	0.035
Farm business	−0.0126945	0.0532269	−0.24	0.811
Constant	0.9374655	0.305284	3.07	0.002
/athrho	−0.455089	0.1368257	−3.33	0.001
/lnsigma	−0.8567397	0.0151515	−56.54	0.000
rho	−0.4260732	0.1119866		
sigma	0.424544	0.0064325		
Number of observations	2178			
Wald Chi square (15)	101.75			0.000
Log pseudo likelihood	−1767.9601			
Wald test of exogeneity (Chi Square)	11.06			0.000

## Data Availability

The World Bank granted the permission to download the data from https://microdata.worldbank.org/index.php/catalog/3716 (accessed on 1 May 2021). However, registered users of the data do not have the permission to share it with any third party.
